# Myocardial Metabolism in Heart Failure

**DOI:** 10.1007/s11897-023-00589-y

**Published:** 2023-02-17

**Authors:** Sher May Ng, Stefan Neubauer, Oliver J Rider

**Affiliations:** 1 Department of Medicine, University of Oxford Centre for Clinical Magnetic Resonance Research, Oxford, UK; 2 Department of Cardiology, John Radcliffe Hospital, Oxford University Hospitals NHS Foundation Trust, Oxford, UK

**Keywords:** Myocardial metabolism, Heart failure, Substrate utilisation, Metabolic inflexibility

## Abstract

**Purpose of Review:**

Myocardial metabolism is intricately linked to cardiac function. Perturbations of cardiac energy metabolism result in an energy-starved heart and the development of contractile dysfunction. In this review, we discuss alterations in myocardial energy supply, transcriptional changes in response to different energy demands, and mitochondrial function in the development of heart failure.

**Recent Findings:**

Recent studies on substrate modulation through modifying energy substrate supply have shown cardioprotective properties. In addition, large cardiovascular outcome trials of anti-diabetic agents have demonstrated prognostic benefit, suggesting the importance of myocardial metabolism in cardiac function.

**Summary:**

Understanding molecular and transcriptional controls of cardiac metabolism promises new research avenues for metabolic treatment targets. Future studies assessing the impact of substrate modulation on cardiac energetic status and function will better inform development of metabolic therapies.

## Introduction

A salient feature of the heart is its metabolic flexibility [1], allowing it to efficiently adapt to varying ATP demands through utilisation of multiple energy substrates such as fatty acids, glucose, lactate, ketones, and amino acids. This is achieved through a complex network of interacting metabolic pathways involving each class of energy substrate, which in turn is capable of remodelling in chronic pathophysiological conditions [2]. As cardiac function is intricately linked to its metabolism, alterations in metabolic pathways can lead to morphological and functional changes. This is seen most clearly in heart failure, where metabolic inflexibility and energetic deficit are associated with maladaptive metabolic remodelling [3, 4].

This review aims to provide an overview of cardiac metabolism in heart failure at its three most important stages: [1] substrate utilisation, [2] ATP generation, and [3] ATP transport and stores. The role of mitochondrial function in the development of heart failure, the importance of transcriptional and post-translational modification in the regulation of cardiac metabolism, and an overview of how perturbations in this tightly regulated system can lead to the development of cardiac dysfunction are included.

### Cardiac Metabolism in the Normal Heart

Step 1: Substrate Uptake and Metabolism

The first stage of cardiac metabolism is substrate delivery, uptake, and generation of acetyl-CoA to facilitate entry into the tricarboxylic acid (TCA) cycle [5, 6•]. Under basal conditions, the majority of acetyl-CoA formation is from long-chain fatty acyl-coenzyme A (60–90%) and pyruvate (10–40%, derived equally from glycolysis and lactate) [7, 8]. Alternative sources such as ketone bodies and amino acids form a minor source of ATP generation under normal physiological conditions.

**Fig. 1:**
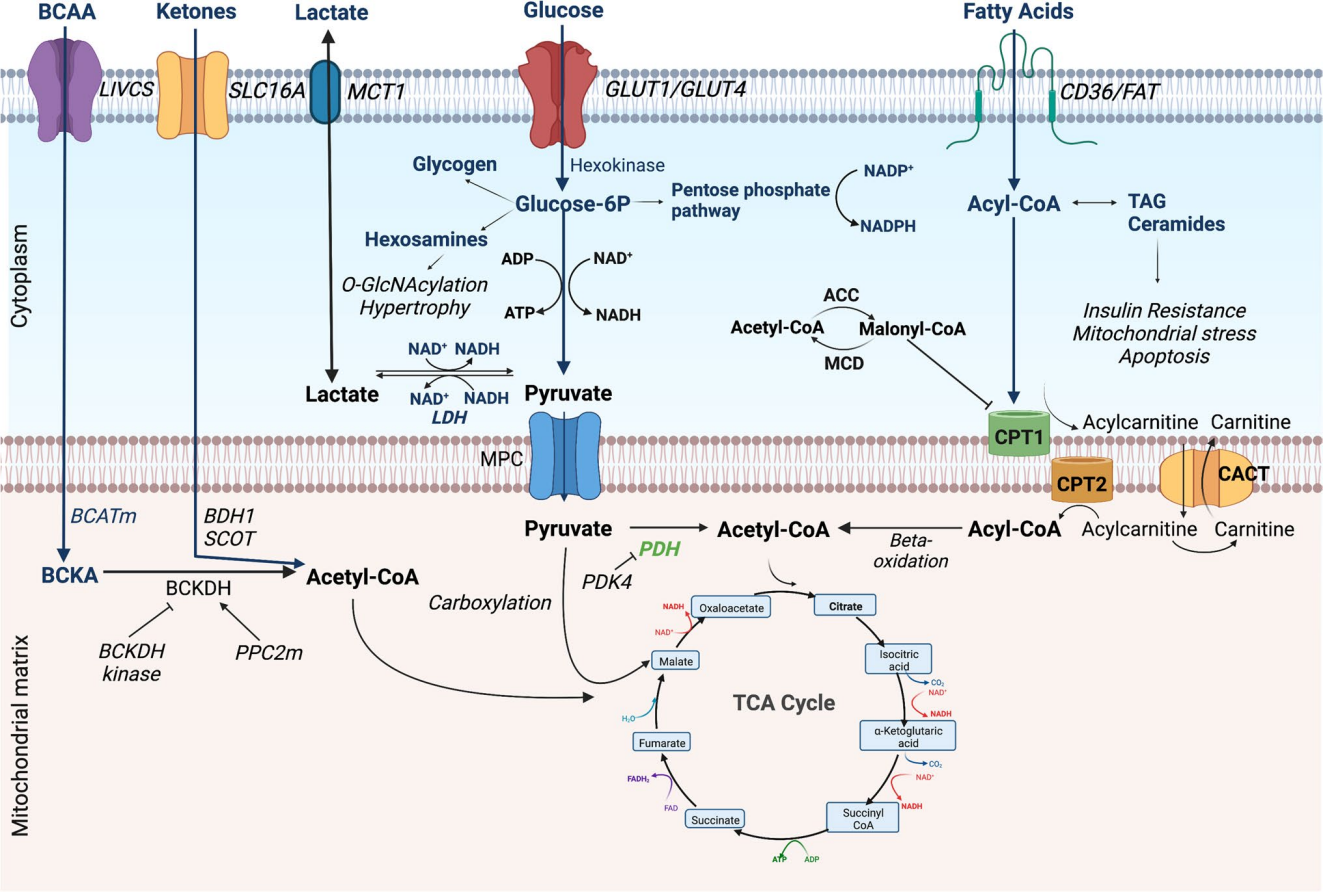
Overview of energy metabolism pathways in a normal heart. Fatty acids are transported into the cardiomyocyte via CD36 and fatty acid transporter protein (FAT) and undergo esterification to form fatty acyl-CoA. The acyl group is transferred to carnitine by carnitine palmitoyltransferase 1 (CPT-1) to form long-chain acylcarnitine and is transported into the mitochondria where it is converted back to fatty acyl-CoA by CPT-2. The fatty acyl-CoA then undergoes beta-oxidation to form acetyl-CoA. The activity of CPT1 is regulated by malonyl-CoA which is formed through carboxylation of acetyl-CoA by acetyl-CoA carboxylase (ACC) and reverted to acetyl-CoA by malonyl-CoA decarboxylase (MCD). The activity of ACC is regulated by phosphorylation via energy sensors such as AMP-activated protein kinase (AMPK). Glucose enters the cardiomyocyte through glucose-transporters-1 or 4 (GLUT-1, GLUT-4). Free glucose is phosphorylated into glucose-6-phosphate (glucose-6P), which can enter different pathways such as glycolysis, pentose phosphate pathway, or the hexosamine biosynthesis pathway. Glycolysis remains the main pathway, producing pyruvate, reduced nicotinamide adenine dinucleotide (NADH), and a small amount of ATP [122]. Lactate is transported into the cardiomyocytes via the monocarboxylic acid transporter (MCT1) and converted into pyruvate by lactate dehydrogenase (LDH), a process consuming reduced nicotinamide adenine dinucleotide (NADH). Pyruvate enters the mitochondria via the mitochondrial pyruvate carrier (MPC) and is converted into acetyl-CoA by pyruvate dehydrogenase (PDH). PDH is inhibited by phosphorylation through pyruvate dehydrogenase kinase isoenzyme 4 (PDK4). Pyruvate can also undergo carboxylation to form malate or oxaloacetate to replenish the TCA cycle (anaplerosis) [123]. Ketones are taken up into the cardiomyocyte by monocarboxylate transporter 1 (encoded by SLC16A gene) and undergo a series of reactions mediated by β-hydroxybutyrate dehydrogenase 1 (BDH1), succinyl-CoA:3 oxoacid-CoA transferase (SCOT), and acetyl-CoA acyltransferase (ACAT1) to form acetyl-CoA. Branched-chain amino acids (BCAA) are transported into the cardiomyocyte via the branched-chain amino acid:cation symporter family (LIVCS). It is converted into branched-chain ketoacids (BCKA) by the mitochondrial branched-chain aminotransferase (BCATm) and forms acetyl-CoA and succinyl-CoA via oxidative decarboxylation by branched-chain alpha-ketoacid dehydrogenase (BCKDH). The activity of BCKDH is regulated via phosphorylation by BCKDH kinase and dephosphorylation through protein phosphatase C2m (PPC2m). CACT, carnitine-acylcarnitine translocase

The selection of energy substrate for ATP production depends on substrate availability, energy demands, and the prevailing metabolic and hormonal conditions. Each energy substrate pathway is regulated by rate-limiting reactions, which in return respond to feedback or feedforward loops, and end-product inhibition as well as energy ‘sensors’ such as AMP-activated protein kinase (AMPK). One example of this feedback inhibition is demonstrated by the reciprocal relationship between myocardial fatty acid and glucose metabolism (Randle cycle), where accumulation of acetyl-CoA results in the activation of pyruvate dehydrogenase kinase (PDK4) and inhibition of pyruvate oxidation [9]. Figure 1 summarises the metabolic pathways corresponding to each energy substrate of the myocardium [7, 10].

Step 2: ATP Generation via Oxidative Phosphorylation

NADH and FADH_2_ produced from the TCA cycle and glycolysis donate electrons to the respiratory chain, releasing free energy which is used to create an electrochemical proton gradient across the mitochondrial inner membrane. The gradient drives ATP production by F_1_/F_0_ ATP synthase [11]. Under aerobic conditions, this contributes to >95% of ATP produced. The rate of oxidative phosphorylation is regulated by ADP and mitochondrial Ca^2+^ homeostasis. In a state of increased ATP demand, there is an increase in cytosolic Ca^2+^ flux, promoting mitochondrial Ca^2+^ uptake. This is crucial in regenerating reduced pyridine nucleotides through increased TCA cycle activity [12].

Step 3: ATP Shuttling and Buffering: the Creatine Kinase System

The final step in cardiac energy metabolism involves the shuttling of ATP to cellular regions of high ATP demand. This link between energy production and energy utilisation is provided by the creatine kinase (CK) system. The CK reaction consists of a phosphate donor (phosphocreatine, PCr), a phosphate acceptor (creatine, Cr), and the catalytic enzyme, CK, as follows:

$$\mathrm{MgADP}^-+\mathrm{PCr^{2-}}\rightleftharpoons\mathrm{MgATP^{2-}}+\mathrm{creatine}$$

This generates phosphocreatine (PCr) which is comparatively smaller and less polar than ATP, allowing rapid diffusion from the mitochondria into the cytosol. The reverse reaction is catalysed by the cytosolic CK, transferring the phosphoryl group back to ADP [13]. As such, the CK system buffers ATP levels in settings of fluctuating demand [14]. As the CK equilibrium constant favours ATP production by nearly 100-fold, this maintains ATP levels in most situations except in advanced heart failure [15, 16].

#### Energy ‘Sensors’

AMP-activated protein kinase (AMPK) and mechanistic target of rapamycin (mTOR) are evolutionarily conserved kinases that regulate cellular metabolism through sensing energy and nutrient levels. The activation of AMPK is dependent on intracellular energy levels, specifically an increased AMP/ATP ratio, as well as upstream kinases such as liver kinase B1 (LKB1) and calcium/calmodulin-dependent protein kinase (CAMKK) [17, 18]. AMPK activation promotes catabolic pathways such as glucose and fatty acid (FA) oxidation and inhibits anabolic processes (e.g. protein synthesis) mediated by mTOR signalling. AMPK also regulates signalling pathways controlling mitochondrial biogenesis and autophagy [19].

**Fig. 2:**
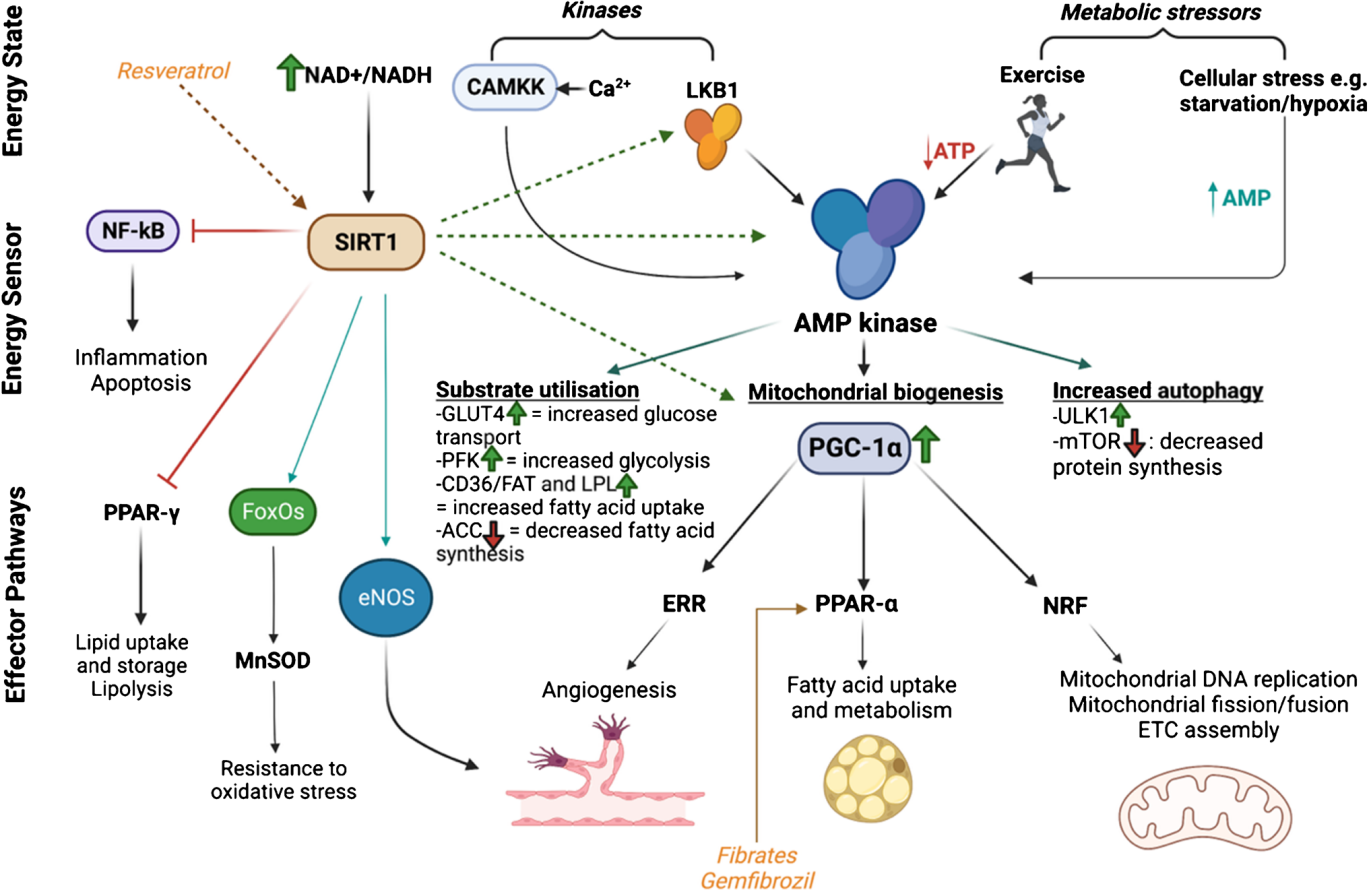
Overview of AMP-kinase, sirtuin-1 (SIRT1), peroxisome proliferator-activated receptors (PPARs), and PPAR-gamma coactivator (PGC-1α) pathways. The mTOR signalling pathway regulates multiple anabolic cellular processes, including protein synthesis, cellular proliferation, cell metabolism, and autophagy. CAMKK, calcium/calmodulin-dependent protein kinase 2; LKB1, liver kinase B1; NF-kB, nuclear factor kappa B; MnSOD, manganese superoxide dismutase; eNOS, endothelial nitric oxide synthase; ERR, oestrogen-related receptors; NRF, nuclear-related factor; ULK1, Unc-51-like autophagy activating kinase; mTOR, mechanistic target of rapamycin, PFK, phosphofructokinase

Energy ‘sensing’ and control of mitochondrial metabolism are also achieved through post-translational modification. The sirtuin family (SIRT), a group of NAD^+^-dependent histone deacetylase (HDAC), in particular the nuclear SIRT1 and mitochondrial SIRT3 regulate multiple cellular pathways (Fig. 2) through interactions with protein kinases such as LKB1, AMPK, and PGC1α [20]. More recently, SIRT1 activation with resveratrol reversed cardiac remodelling and improved function in an animal model of diabetic cardiomyopathy [21] and dilated cardiomyopathy [22].

#### Transcriptional Regulation of Metabolic Pathways

Central to metabolic transcriptional remodelling in heart failure are the peroxisome proliferator-activated receptors (PPARs), a network of nuclear transcription factors crucial for fatty acid metabolism and mitochondrial biogenesis. Of the three isoforms (α, δ, and γ), PPARα and PPARδ are highly expressed within cardiac myocytes. PPARα increases fatty acid uptake, oxidation, and ketogenesis [23], and its overexpression produces metabolic phenotypes mimicking insulin resistance [24, 25]. Conversely, PPARδ plays a balanced role in increasing both FA and glucose utilisation, as well as regulating the reactive oxygen species (ROS) scavenging system [26, 27]. PPARγ is expressed at very low levels in cardiomyocytes and regulates intracellular lipid trafficking [28].

A pivotal transcriptional coregulator within cardiomyocytes is the PPAR-gamma coactivator (PGC-1α) which responds to a wide range of stimuli including starvation, exercise, and change in substrate availability. The downstream effector pathways of PGC1α are summarised in Fig. 2 and include PPARα, ERRα, and nuclear respiratory factor 1 (NRF1) [29].

### Cardiac Metabolism in Heart Failure

Heart failure is associated with derangement in all three fundamental steps of energy generation. Many single-gene transgenic, gain- and loss-of-function animal models have demonstrated a wide array of altered substrate utilisation with conflicting outcomes on contractile function and remodelling [1, 5, 6•]. This could reflect the differing pathophysiology of cardiac diseases, duration, and severity of disease. The main questions remain - is the metabolic remodelling an adaptive or maladaptive response to external stimuli? Is an altered energetic state the cause or consequence of heart disease?

## Substrate Utilisation in Heart Failure

Metabolic remodelling and the ‘loss of metabolic flexibility’ are believed to antedate, initiate, and sustain structural and functional remodelling in the development of heart failure. Specifically, reversion to foetal gene expression, impairment of FA oxidation (FAO) capacity, and insufficient compensatory increases in glucose metabolism are thought to underlie the transition to heart failure.

### Fatty Acid Metabolism

Animal models of altered fatty acid metabolism in the pathophysiology of heart failure have been inconsistent, showing fatty acid oxidation is either decreased [30, 31], unchanged [32, 33], or increased [34]. This may be due to differences between animal models, disease aetiology, and stage of disease. Whether these changes represent an adaptive or maladaptive response remains unresolved.

A key observation in pathological hypertrophy and heart failure is the downregulation of PPAR α, an important transcriptional regulator of lipid metabolism [35]. PPARα knockout (KO) mice demonstrate severely impaired FAO and increased cardiac glucose oxidation but fail to maintain contractile performance at high workload with increased myocardial fibrosis [36, 37]. Conversely, increased PPARα expression in transgenic mice increases fatty acid uptake, assimilating a phenotype similar to that of diabetic or obese cardiomyopathy [38]. However, increasing both glucose and fatty acid metabolism, thus overall myocardial oxidative metabolism, through PPARβ/δ overexpression appears cardioprotective against mechanical stress [39].

Dysregulation of specific pathways of fatty acid metabolism also appears to have deleterious effects. Interruption in fatty acid uptake alone through deletion of CD36 or carnitine palmitoyltransferase-1 (CPT-1) exacerbated cardiac hypertrophy and cardiac dysfunction in animal models [40, 41]. Aberration in fatty acid uptake and its association with pathological hypertrophy and heart failure is further supported by the observation of a high prevalence of CD36 deficiency in patients with dilated cardiomyopathy [42].

Despite this, clinical trials suggest further inhibition of fatty acid oxidation in order to recouple glycolysis to glucose oxidation may be beneficial. These include partial fatty acid oxidation inhibitors such as trimetazidine [43] and ranolazine [44] as well as CPT-1 inhibitors, e.g. perhexiline [45]. Of note, these agents may exert its beneficial effects through pathways beyond that of metabolic alteration (e.g. inhibition of late inward sodium channels by ranolazine) and these studies remain limited by their small sample size. Some animal models of malonyl-CoA inhibition have also shown promise but this has not been studied in humans [46].

The effects of increasing FAO on cardiac remodelling have also been investigated. The specific upregulation of mitochondrial FAO through deletion of ACC2 appears protective against cardiac hypertrophy in ACC2-KO mice [47]. While animal models of PPARα agonism have shown variable results [48], there have been no clinical trials specifically studying the effect of PPARα modulation on heart failure outcomes. However, post hoc analyses of a large dyslipidaemia and diabetes trial have shown reduction in heart failure hospitalisation in patients treated with PPARα agonists, e.g. fenofibrate [49, 50•].

Overall, these studies suggest the importance of balancing fatty acid availability, uptake, and oxidation in maintaining normal cardiac function. Certainly, preservation of myocardial capacity for FAO appears cardioprotective to a degree, especially in context of haemodynamic stress. Thus, over-reliance on one energy substrate regardless of which substrate this may be and the inability to utilise another would seem a maladaptive response in heart failure.

### Glucose Metabolism

The majority of animal models of a failing heart, whether subjected to pressure-overload or pacing-induced cardiomyopathy, have demonstrated the uncoupling between glycolysis and glucose oxidation. The shift towards glucose utilisation in context of the failing heart is thought to improve myocardial ‘efficiency’ by producing more ATP per molecule of oxygen consumed [51].

However, considering that fatty acids remain a very effective carbon source for high volume of ATP production and are abundantly available, does increased glucose reliance (and subsequently impaired FAO) contribute to the development of cardiac dysfunction? PPARα nulled hearts fail to maintain myocardial energetic demands in the presence of high cardiac workload, developing cardiomyopathy at old age. This is interestingly reversed through the overexpression of GLUT1[37], proposing a theory that the inherent capacity of the adult myocardium to increase glucose oxidation in context of decreased FAO capacity may not provide sufficient ATP under stress. In addition, the unmatched rise in glycolytic rates cause build-up of glycolysis-derived protons, intracellular acidosis, and decreased cardiac contractility due to inhibition of slow Ca^2+^ current [52].

Therefore, it is reasonable to propose that increasing both glucose uptake and oxidation capacity may improve myocardial energetics. Studies of glucose-insulin-potassium (GIK) infusions and glucagon-like peptide (GLP-1) agonists which aim to increase insulin secretion, sensitivity, and glucose uptake have suggested an improvement in cardiac function in patients with ischaemic cardiomyopathy in settings of an acute myocardial infarction [53, 54]. Similarly, inhibition of PDH kinase (PDHK) with dichloroacetate (DCA) appears to improve haemodynamic status (e.g. stroke volume and myocardial work) in patients with coronary artery disease and congestive heart failure [55, 56].

#### Carbohydrate vs. Fatty Acid

Animal models mimicking metabolic shifts towards a specific substrate, e.g. glucose only or fatty acid only have provided further insight. Cardiac-specific overexpression of PDK4 leading to increased FAO and decreased glucose metabolism was not detrimental to cardiac function following an ischaemic insult [57], suggesting increased FAO alone may not be key to abnormal cardiac remodelling.

Substrate modification on GLUT-1 transgenic mice shed further light on the importance of preserving a balanced metabolic profile. When exposed to a high-fat diet, GLUT-1 transgenic mice were observed to maintain a high glucose oxidation rate (unlike control animals) suggesting longer-term transcriptional downregulation of fatty acid metabolism in these transgenic mice. Importantly, this was accompanied by increased oxidative stress and development of contractile dysfunction [58].

This proposes that the focus of therapeutic targets of metabolism should be that of sustaining flexibility of substrate utilisation, rather than ‘preferencing’ one over another. More recently, the cardioprotective role of substrate manipulation has been explored in surgical post-coronary revascularisation. Intralipid infusion post-sternotomy as a pre-conditioning agent was associated with reduced need for high-dose inotropic support, earlier normalisation of serum troponin levels, and higher values of cardiac index measured by invasive cardiac monitoring [59]. These effects are believed to be mediated by phosphorylation of glycogen synthase kinase-3β (GSK-3β) and delaying of the opening of the mitochondrial permeability transition pore (mPTP) associated with cellular apoptosis [60]. The effect of substrate modulation and metabolic flexibility has been further studied with magnetic resonance spectroscopy, where intralipid infusion was seen to improve myocardial energetics (PCr/ATP ratio) and cardiac contractility in patients with established non-ischaemic dilated cardiomyopathy [61]. This represents an exciting new approach to manipulating metabolism for improved cardiac function.

#### Ketone Body Metabolism

Ketone bodies (β-hydroxybutyrate (BOHB) and acetoacetate) are produced by the liver during periods of fasting. Upon entering cardiomyocytes and the mitochondrial matrix, ketone bodies form acetyl-CoA through a series of reactions catalysed by BOHB dehydrogenase (BDH1), succinyl-CoA:3-oxoacid-CoA-transferase (SCOT), and acetyl-CoA acetyltransferase (ACAT1) within the mitochondria. Under normal circumstances, ketone bodies account for 5–10% of the myocardial energy substrate metabolism [62]. Serum ketone levels are the major determinant of myocardial ketone body oxidation rate.

Circulating ketone levels are reportedly increased in patients with heart failure [63]. In parallel, BDH1 and SCOT expressions are upregulated in animal and human models of heart failure, supporting the notion of increased reliance on ketone body utilisation in a diseased state [64]. However, the same question applies—is this increased reliance an adaptive or maladaptive response? Gene-targeted mouse models suggest that increased myocardial ketone body oxidation is adaptive; SCOT-KO mice exhibit accelerated pathological remodelling, mitochondrial dysfunction, and exacerbated cardiac dysfunction in context of pressure-overload [65]. Conversely, cardiac-specific BDH1 overexpression had enhanced antioxidant enzyme expression and was resistant to contractile dysfunction when subjected to pressure-overload stimuli [66].

Nevertheless, ketone body utilisation has a reciprocal relationship with glucose and fatty acid metabolism. Ketone body infusion in healthy human volunteers was associated with increased myocardial blood flow but decreased myocardial glucose uptake on positron emission tomography, an effect due to interrupted GLUT4 translocation [67, 68]. BOHB also appears to inhibit myocardial fatty acid uptake, through a mechanism independent of malonyl-CoA levels [69].

The consequences of this inhibitory effect of ketone bodies on myocardial glucose and fatty acid uptake require further characterisation. However, stimulating ketone oxidation appears to improve cardiac function. Increasing ketone body availability through either intravenous ketone infusions or oral ketone esters has beneficial impact on cardiac haemodynamics and function measured by LVEF [70•, 71]. Sodium-glucose-linked transporter 2 (SGLT2i) inhibitors may exert cardioprotective effects through increasing circulating ketone bodies to an ‘energy-starved’ failing heart [72]. This hypothesis is supported by a non-diabetic porcine heart failure model where empaglifozin led to a switch towards ketone body and fatty acid utilisation and amelioration of adverse cardiac remodelling [73•].

#### Branched-Chain Amino Acids (BCAA) Metabolism

Branched-chain amino acids (BCAA) such as leucine, isoleucine, and valine account for <2% of ATP production in a normal heart under physiological conditions [74]. These enter the cell through specialised amino acid transporters regulated by substrate availability [75]. BCAAs undergo transamination (by the mitochondrial branched-chain amino-transaminase, BCATm) to form corresponding branched-chain alpha-ketoacids (BCKA). The rate-limiting step of BCAA metabolism is the subsequent oxidative decarboxylation of BCKAs by the branched-chain alpha-keto acids dehydrogenase complex (BCKDH) to form either acetyl-CoA for the TCA cycle or succinyl-CoA for anaplerosis. This step is regulated by phosphorylation (inhibition) and dephosphorylation (activation) of BCKDH by either BCKDH kinase or protein phosphatase C2m (PPC2m), respectively [76].

Animal and human models of the failing heart demonstrate impaired BCAA oxidation, increased BCAA and BCKA levels, and decreased expression of enzymes involved in BCAA metabolism [77, 78]. Although less significant in energy generation, accumulation of BCAA and BCKA promotes cardiac hypertrophy through persistent mTOR signalling and cardiac insulin resistance [79, 80, 81]. In addition, accumulation of BCKA is believed to disrupt mitochondrial redox homeostasis through significantly increased superoxide production. Importantly, these effects are reversed with enhanced BCAA oxidation and inhibition of mTOR signalling with rapamycin in mouse models [77].

These findings have stimulated research interests in developing therapeutic strategies targeting BCAA metabolism in heart failure. Inhibition or stimulation of BCATm is limited by the unintended consequence of preferentially increasing BCAA or BCKA respectively. Inhibition of BCKDH kinase with BT2 (3,6-dichlorobenzo[b]thiophene-2-carboxylic acid), thus decreased BCAA and BCKA levels, in a pressure-overload mouse model ameliorated negative cardiac remodelling and dysfunction; however, there are no human trials to date [82]. More recently, dietary interventions in animal and human models suggest the potential of improving BCAA metabolism and insulin sensitivity, providing yet another metabolic target for heart failure treatment [83, 84].

## Mitochondrial Dysfunction in Heart Failure

**Fig. 3:**
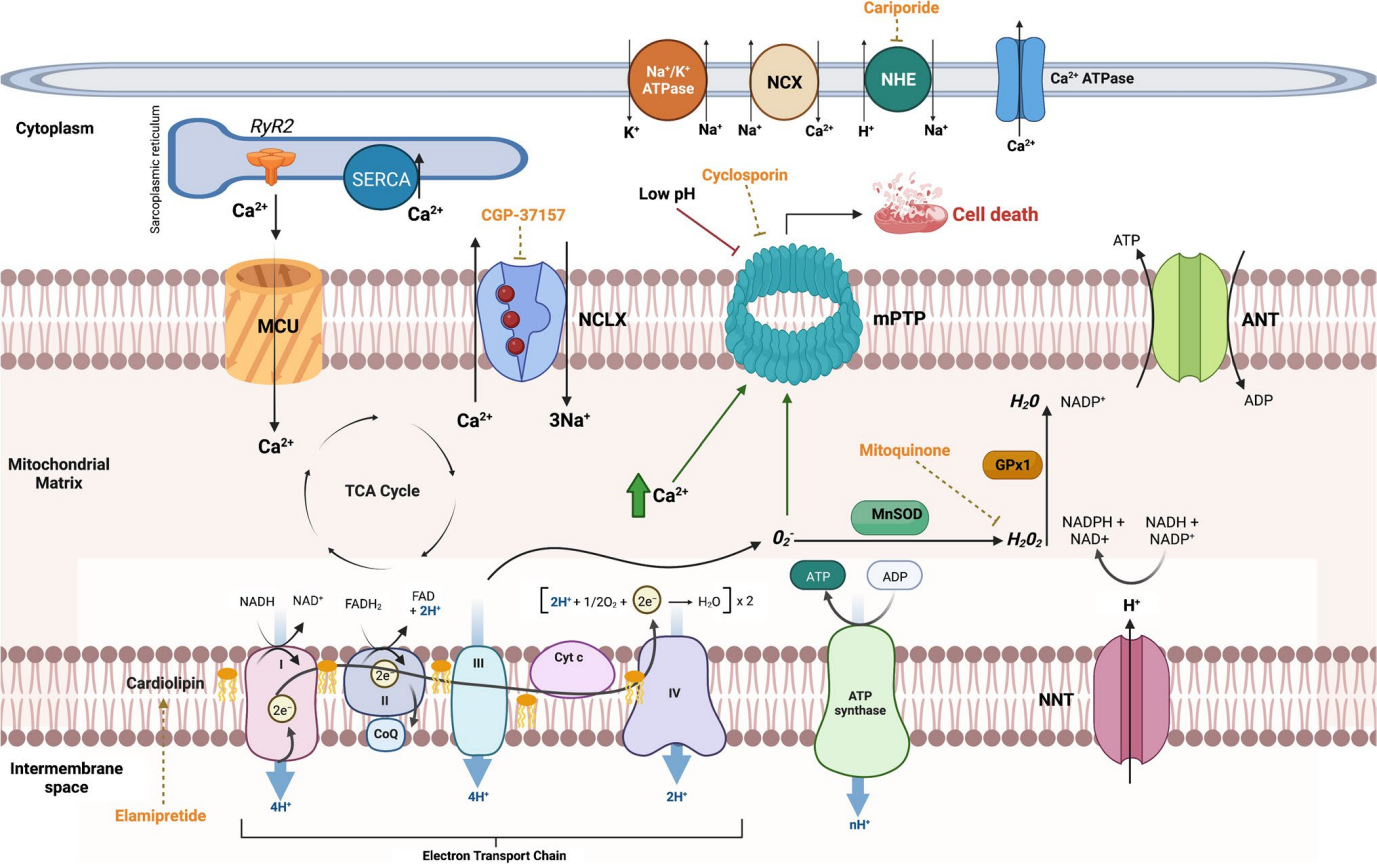
Overview of mitochondrial function including ATP generation, redox signalling, and calcium handling. In a healthy cardiomyocyte, the ryanodine receptor (RyR2) of the sarcoplasmic reticulum (SR) is in close proximity with the mitochondria, where the mitochondrial calcium uniporter (MCU) is located. This facilitates a specific concentration of free Ca^2+^ in the SR-mitochondria interface to enable mitochondrial uptake of cytosolic Ca^2+^, a process that sustains mitochondrial metabolism for cardiomyocyte contraction. Mitochondrial Ca^2+^ efflux is mediated by the mitochondrial Na^+^/Ca^2+^ exchanger (NCLX) located near the sarcoplasmic reticulum Ca^2+^ ATPases (SERCA). Cytosolic Ca^2+^ levels are also regulated by other transporters in the plasma membrane such as the sodium-calcium exchanger (NCX), Ca^2+^ ATPases, and sodium-hydrogen exchanger (NHE). The mitochondrial permeability transition pore (mPTP) located in the inner mitochondrial membrane is normally closed and opens when stimulated by mitochondrial Ca^2+^ overload—this results in mitochondrial osmotic swelling and dissipation of the mitochondrial inner membrane potential, ultimately resulting in cell death. Reactive oxygen species such as superoxide (O_2_^−^) are produced at complex I and III of the electron transport chain and are converted to H_2_O_2_ by manganese superoxide dismutase (MnSOD). Mitochondrial H_2_O_2_ is converted to H_2_O by catalase and mitochondrial-localised glutathione peroxidases (GPx1), consuming reducing equivalents such as NADPH. ANT, adenine nucleotide translocase; NNT, nicotinamide nucleotide transhydrogenase. *Figures 1, 2, and 3 were created with BioRender.com

In addition to a loss of metabolic flexibility, heart failure is associated with derangement in mitochondrial function resulting in mechano-energetic uncoupling; these include abnormal mitochondrial structure and function, increased production of reactive oxygen species (ROS), altered mitochondrial ion homeostasis, and impairment in ATP generation. Figure 3 provides an overview of mitochondrial function.

## Abnormal Mitochondrial Structure and Function

Mitochondria in heart failure undergo several structural changes [85, 86] that reduce mitochondrial respiratory capacity in animal and human models of heart failure. In particular, the expression of a key phospholipid within the inner mitochondrial membrane, cardiolipin, appears to be reduced in heart failure [87, 88].

Cardiolipin functions as a key co-factor for mitochondrial transport proteins, including the respiratory supercomplexes (complex I, III, IV and V). and plays a role in the retention of cytochrome c, a key regulator of cellular apoptosis within the inner mitochondrial membrane. ROS-mediated oxidation of cardiolipin results in release of cytochrome c into the cytoplasm and activation of caspase-3. The importance of cardiolipin is exemplified in genetic disorders that affect cardiolipin synthesis such as Barth syndrome (BTHS) and dilated cardiomyopathy with ataxia (DCMA) [89]. Cardiolipin levels in heart failure are also reduced, leading to increased production of ROS, peroxidation of cardiolipin, mitochondrial dysfunction, and cellular death [90].

## Increased ROS Production

ROS production is low under physiological conditions and kept in check by endogenous scavenging systems. Mitochondria in the failing heart are associated with an increased production of ROS, beyond the capacity of the endogenous ROS scavenging system [91, 92]. Elevated mitochondrial ROS production results in mitochondrial DNA damage, defect in the electron transport chain ultimately triggering cell death cascades. As such, targeting ROS with antioxidant therapies would seem a promising approach in treatment of cardiovascular disease. Nevertheless, trials with non-specific antioxidants such as vitamin E had no effect on major adverse cardiovascular events, or indeed hospitalisation for heart failure [93]. Possible barriers to this include the untargeted treatment as well as lack of cell permeability. Although targeted antioxidants such as mitoquinone (MitoQ) have demonstrated cardioprotective effects in animal models of pressure-overload and ischaemia-reperfusion injury, this has not been trialled in patients with heart failure to date [94, 95].

## Altered Calcium Handling

Mitochondrial calcium handling is closely linked to its role in excitation-contraction coupling, ATP synthesis and cellular death. The increased demand for ATP in conditions of high workload is facilitated by a matched rise in the rate of ADP phosphorylation through acceleration in the TCA cycle. This is achieved by accumulation of calcium (Ca^2+^) within the mitochondrial matrix. Mitochondria also function as a reservoir and are able to accumulate a large amount of Ca^2+^ [96]. This is regulated by the close proximity between the mitochondrial Ca^2+^ uniporter protein (MCU) in the inner mitochondrial membrane (IMM) and the sarcoplasmic reticulum (SR), as well as the mitochondrial Na^+^/Ca^2+^ exchanger (NCLX), which facilitates Ca^2+^ efflux [97].

In a failing heart, the function of these membrane-bound pumps for calcium handling is affected due to its energy and ROS dependence. Cellular calcium handling is deranged due to increased ROS production and intracellular sodium levels in heart failure [98, 99]. Reduced expression of cardiac SR calcium ATPase (SERCA2a) also contributes to increased end-diastolic cytosolic calcium level and prolongation of calcium transient during diastole, causing impaired diastolic relaxation in heart failure with preserved ejection fraction (HFpEF) [100].

Targeting therapies that augment mitochondrial Ca^2+^ uptake or prevent Ca^2+^ extrusion may prove beneficial considering the role of Ca^2+^ in replenishing the endogenous ROS scavenging system. Selective NCLX inhibition with CGP-37157 in a guinea pig model of heart failure and sudden cardiac death preserved cardiac contractility and prevented fatal arrhythmias [101]. To date, this has not been trialled in humans. A selective inhibitor of NHE type I, cariporide in ischaemia/reperfusion injury, was unfortunately associated with increased mortality in patients undergoing high-risk coronary artery bypass surgery, despite reducing the rate of myocardial injury [102, 103].

Conversely, mitochondrial Ca^2+^ overload precipitates the opening of a high-conductance channel within the IMM—the mitochondrial permeability transition pore (MPTP). Opening of MPTP is associated with loss of IMM potential (and thus uncoupling of oxidative phosphorylation), mitochondrial dysfunction, and subsequent cellular death. Cardiomyocytes from animal models of heart failure demonstrate an increased tendency for mPTP opening and decreased mitochondrial membrane potential, with inhibition of MPTP using cyclosporin ameliorating mitochondrial dysfunction [104].

## Abnormalities in Electron Transport Chain (ETC)

Impaired oxidative phosphorylation secondary to ETC abnormalities has also been reported, including reduced number and function of the respiratory complexes [105, 106] and reduced coenzyme Q (CoQ) levels. CoQ functions as a redox-cycling coenzyme within the ETC, with the ability to accept or donate an electron depending on its redox potential. In line with this, the Q-SYMBIO trial showed CoQ supplementation led to symptomatic improvement and reduced major adverse cardiovascular events in heart failure with reduced ejection fraction [107].

### Energy Buffering and Transfer in Heart Failure

The creatine kinase (CK) system is a crucial component of the metabolic machinery, especially in organ tissues with high and intermittent energy fluctuations such as the heart. It serves as a ‘temporal’ energy buffer, maintaining adequate ATP concentration according to energy demand, and a ‘spatial’ buffer tightly coupling ATP-producing and consuming processes. Creatine (Cr) is absorbed from dietary sources and synthesised endogenously in a two-stage process. Transport of Cr into myocytes is mediated by a Na^+^/Cl^-^ dependent transporter (CrT; SLC6A8) and is largely regulated by substrate availability and cellular metabolite state [108].

A reduction in PCr/ATP ratio is well-documented across a range of cardiac pathology [109]. Additionally, a lower PCr/ATP ratio is correlated with a higher NYHA class, lower left ventricular ejection fraction, and a worse prognosis [109]. The initial decrease in PCr/ATP reflects a decrease in PCr levels as ATP concentrations are seen to decrease only at advanced stages of HF [110]. Of note, a pseudonormalised PCr/ATP ratio may be achieved when ATP is depleted at later stages of disease, thus underestimating changes in myocardial energetic state [111].

The rightward shift in CK equilibrium (towards ATP generation) in circumstances of chronically increased workload and a reduction in total myocardial creatine content in a failing heart likely contribute to the decreased PCr/ATP ratio [112]. Creatine pool size is positively correlated with left ventricular ejection fraction in dilated cardiomyopathy and inversely related to plasma NTproBNP levels [113]. Whether creatine deficiency (and thus decreased PCr) contributes to the development of heart failure, however remains unclear.

Animal models on the causal role of a dysregulated CK system in chronic heart failure remain conflicting. Reduced creatine levels (either pharmacologically through beta-guanidinopropionate or genetic knockout of guanidinoacetate methyltransferase, GAMT) and loss of CK activity in models of chronic heart failure have little consequence on LV function or survival [114]. Nevertheless, a functioning CK system remains important for preserved contractile reserve in context of acute insults or increased workload such as myocardial infarction, although not at rest [115–117]. Indeed, overexpression of plasma membrane CrT in a mouse model of ischaemia/reperfusion injury suggests increased myocardial creatinine levels protect against myocardial necrosis and adverse LV remodelling, supporting the role of improved energy reserve particularly in acute stress [118].

Importantly, some evidence has suggested augmentation of the CK system is cardioprotective in ischaemia/reperfusion injury [119] and heart failure [120]. Moreover, myocardial CK flux remained a significant predictor of heart failure outcomes, independent of NYHA class and LVEF suggesting the role of altered ATP kinetics in risk stratification of HF [121]. Further research to better understand the importance of CK system enhancement will help identify novel heart failure therapies.

## Conclusion

Metabolic changes that occur in heart failure are complex. The numerous animal models attempting to explore these are challenged by the ability to accurately recapitulate disease aetiology, duration, and severity, and as such have provided conflicting information. Nevertheless, there is consistency in the description of transcriptional changes leading to altered cardiac metabolism in the failing heart. This ultimately manifests in the energy-starvation hypothesis described in heart failure. Future studies identifying optimal strategies to restore metabolic flexibility will advance the development of targeted metabolic therapy in heart failure.
